# Progress in geochronology: not only timing, but also rate

**DOI:** 10.1093/nsr/nwaf309

**Published:** 2025-08-01

**Authors:** Xian-Hua Li

**Affiliations:** State Key Laboratory of Lithospheric and Environmental Coevolution, Institute of Geology and Geophysics, Chinese Academy of Sciences, China

Timing is everything. Geochronology, utilizing both absolute dating and relative dating methods, provides the time coordinates essential for understanding the solar system’s evolution and Earth's dynamic history. It determines the timing, duration and rate of geological, biological and environmental processes. This information ultimately facilitates establishing causal relationships between different processes, and reconstructing evolutionary history through a series of geological snapshots. While geochronology plays an increasingly vital role in nearly every field of Earth and planetary science, the field itself is rapidly evolving through theoretical and technical advances. A general trend in these advances is the pursuit of dating methods offering higher spatial resolution, higher precision (and accuracy) and higher efficiency, alongside more robust data interpretation. This Special Topic collection features four Reviews and four Perspectives, showcasing recent advances in both geochronological methods and their applications.

Absolute radioisotopic dating methods rely on radioactive decay systems. Different isotopic systems can be used to date materials ranging in age from billions of years to less than a million years old. Numerous chronometers based on decay of both extant and extinct radionuclides have been developed to date the early solar system, specifically the first seven million years after its formation. Among these, the ^26^Al–^26^Mn, ^53^Mn–^53^Cr and ^182^Hf–^182^W extinct radionuclide chronometers and the Pb–Pb chronometer (based on the U–Pb dual decay system) are the mainstream dating tools for early solar system studies [1]. Other commonly used techniques for dating deep-time geological samples include U–Pb [2], Ar–Ar [3] and Re–Os [4] methods. They can also be applied to young samples (e.g. Quaternary) when datable materials are available. To calibrate paleoclimate records preserved in Quaternary carbonates, U–Th and U–Pb dating methods have been tailored to date carbonate samples younger or older than ∼600 ka, respectively [5]. Complementary to absolute dating methods, relative dating methods primarily constrain the durations of geological processes. One such relative method, diffusion chronometry, leverages chemical heterogeneity within crystals or melts to decipher the thermal evolution of magmatic systems [6].

For each radioisotopic dating system, several analytical protocols have been developed to meet different research demands. These protocols can be grouped into bulk analyses and microanalyses. Bulk analyses should be prioritized when high-precision dating results (better than 0.1%, 2 relative standard deviation) are required to resolve geological processes [2]. While microanalyses usually yield less precise dating results, they offer advantages including higher spatial resolution (micron to sub-micron scale), higher efficiency and lower sample consumption. This makes them particularly suited for dating precious samples with limited material [7]. Advances in mass spectrometers, standardized analytical workflows and refined sample pre-treatment strategies over the past decades have facilitated both bulk and microanalytical protocols. Data interpretation is as critical as data acquisition. The mean square weighted deviation (MSWD) is a widely used measure of statistical dispersion in geochronology. Due to steady improvements in analytical precision and throughput across all geochronological techniques, datasets with high MSWD could become more prevalent in the future. Such dispersion should not be feared but quantitatively evaluated [8].

While significant advances have been achieved in geochronology over recent decades, further development is needed to meet the growing demand for high-precision and high-accuracy dating results. Coordinated efforts are required to advance mass spectrometer technology, secure more reference materials (including isotope spikes) and recalibrate decay constants for some commonly used decay systems. Integration of data from different dating methods is crucial to better calibrate the timing and rates of geological events and understand their mechanisms, fostering stronger collaboration between geochronology and other fields of Earth and planetary science.

I would like to thank all authors, reviewers and editorial staff, without whose contribution and help this Special Topic would not be possible.
